# Ultimate Charge Transport Regimes in Doping-Controlled Graphene Laminates: Phonon-Assisted Processes Revealed by the Linear Magnetoresistance

**DOI:** 10.1021/acsnano.4c05512

**Published:** 2024-08-08

**Authors:** Mohsen Moazzami Gudarzi, Sergey Slizovskiy, Boyang Mao, Endre Tovari, Gergo Pinter, David Sanderson, Maryana Asaad, Ying Xiang, Zhiyuan Wang, Jianqiang Guo, Ben F. Spencer, Alexandra Geim, Vladimir I. Fal’ko, Andrey V. Kretinin

**Affiliations:** † Department of Physics and Astronomy, The University of Manchester, Oxford Road, Manchester M13 9PL, U.K.; ‡ Department of Materials, The University of Manchester, Oxford Road, Manchester M13 9PL, U.K.; § Cambridge Graphene Centre, Department of Engineering, University of Cambridge, 9 JJ Thomson Avenue, Cambridge CB3 0FA, U.K.; ∥ Department of Physics, Institute of Physics, Budapest University of Technology and Economics, Műegyetem rkp. 3, H-1111 Budapest, Hungary; ⊥ Department of Physics, Harvard University, Cambridge, Massachusetts 02138, United States; # National Graphene Institute, The University of Manchester, Oxford Road, Manchester M13 9PL, U.K.; ∇ Henry Royce Institute for Advanced Materials, The University of Manchester, Oxford Road, Manchester M13 9PL, U.K.

**Keywords:** solution-processed 2D materials, graphene laminates, linear magnetoresistance, phonon-assisted tunneling, electron−phonon scattering, graphene thermocouples

## Abstract

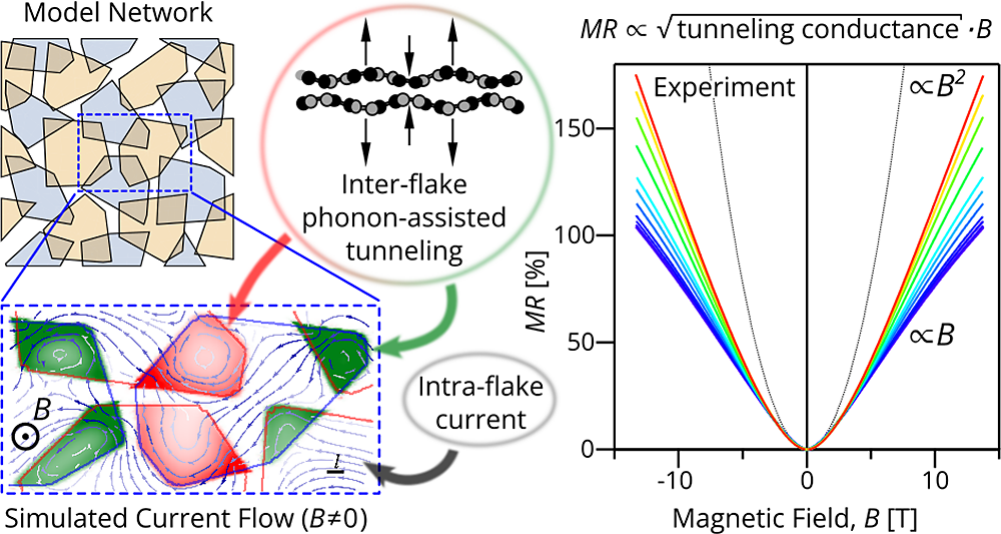

Understanding and controlling the electrical properties of solution-processed 2D materials is key to further printed electronics progress. Here, we demonstrate that the thermolysis of the aromatic intercalants utilized in nanosheet exfoliation for graphene laminates allows for high intrinsic mobility and the simultaneous control of doping type (*n*- and *p*-) and concentration over a wide range. We establish that the intraflake mobility is high by observing a linear magnetoresistance of such solution-processed graphene laminates and using it to devolve the interflake tunneling and intralayer magnetotransport. Consequently, we determine the temperature dependencies of the inter- and intralayer characteristics. The intraflake transport appears to be dominated by electron–phonon scattering processes at temperatures *T* > 20 K, while the interflake transport is governed by phonon-assisted tunneling. In particular, we identify the efficiency of phonon-assisted tunneling as the main limiting factor for electrical conductivity in graphene laminates at room temperature. We also demonstrate a thermoelectric sensitivity of around 50 μV·K^–1^ in a solution-processed metal-free graphene-based thermocouple.

The development of 2D material inks for printed electronics, supported by the expanding and perfected chemical exfoliation routes,^1^ requires deeper insights into the factors limiting the electrical performance of the solution-processed 2D materials films.^2^ The macroscopic models based on a phenomenological circuit theory^3,4^ imply that the overall resistance of a network of weakly coupled conductive nanosheets is defined by the interplay between their intrinsic conductivity and an interfacial “contact” resistance.^5,6^ The mechanisms of electrical conductivity in the printed network of 2D nanoparticles have been recently probed by tracking the temperature dependence of resistivity.^7−11^ For example, an exponential increase of resistivity upon cooling the film points toward thermally activated variable-range hopping transport between small semi-isolated grains.^9,11−13^ However, a variable-range hopping transport scenario hardly applies to a laminate composed of large-area metallic nanosheets with high intrinsic mobility.^10^ Such materials have recently been studied, where the reported high values of optical conductivity increase upon cooling,^8,10,14^ highlighted the role of phonons in electronic transport. At the same time, charge transport across the boundaries of nanosheets remains the main limiting factor for the film conductivity, especially in laminates produced from layered van der Waals materials,^15−20^ which are sensitive to the implemented exfoliation chemistry and postprocessing, relative twist angle between the crystals, residually trapped intercalants, and packing density.^19,21−23^

Here, we study the graphene laminates produced by chlorosulfuric acid-assisted liquid phase exfoliation,^24^ shown to deliver large aspect ratio (∼10^3^) high-quality nanosheets of graphene. In particular, we perform magnetotransport characterization of multiple solution-processed laminates with the same structural properties but different charge carrier types and doping densities controlled by the postprocessing annealing. The analysis of magnetotransport data enables us to devolve the parametric dependences (temperature and carrier density) of the intra- and interflake transport. The application of a strong magnetic field leads to the distortion of the intrananosheet current flow toward their edges, forcing the carrier tunneling between the adjacent flakes to occur near the edges rather than across the whole overlap area, making the intralayer charge transport similar to that in a two-terminal Hall resistor.^25^ In the reported experiment, this crossover was manifested as a transition of the observed magnetoresistance (MR) from a quadratic to linear magnetic field dependence. The quantitative devolution of the measured parametric dependences was made using a mesoscale numerical model applied to a variety of networks composed of weakly coupled high-mobility 2D conductors, with the temperature dependences of the devolved transport parameters pointing toward the dominance of electron–phonon scattering processes in both intraflake conductivity and interflake tunneling. Moreover, the performed XPS, Raman and X-ray diffraction characterization indicated that the only nongraphene chemical present in the laminates was the tetra-sulfonated pyrene (s-Py) molecules introduced during the exfoliation. Using the postprocessing thermolysis for removing the sulfonic groups of the s-Py molecules, we modify both graphene doping and interflake tunneling and optimize the material to achieve a substantial thermoelectric response, producing a highly sensitive all-graphene solution-processed thermocouple.

## Results and Discussion

### Laminates of Large Aspect Ratio Bilayer Graphene Nanosheets

**Figure 1:**
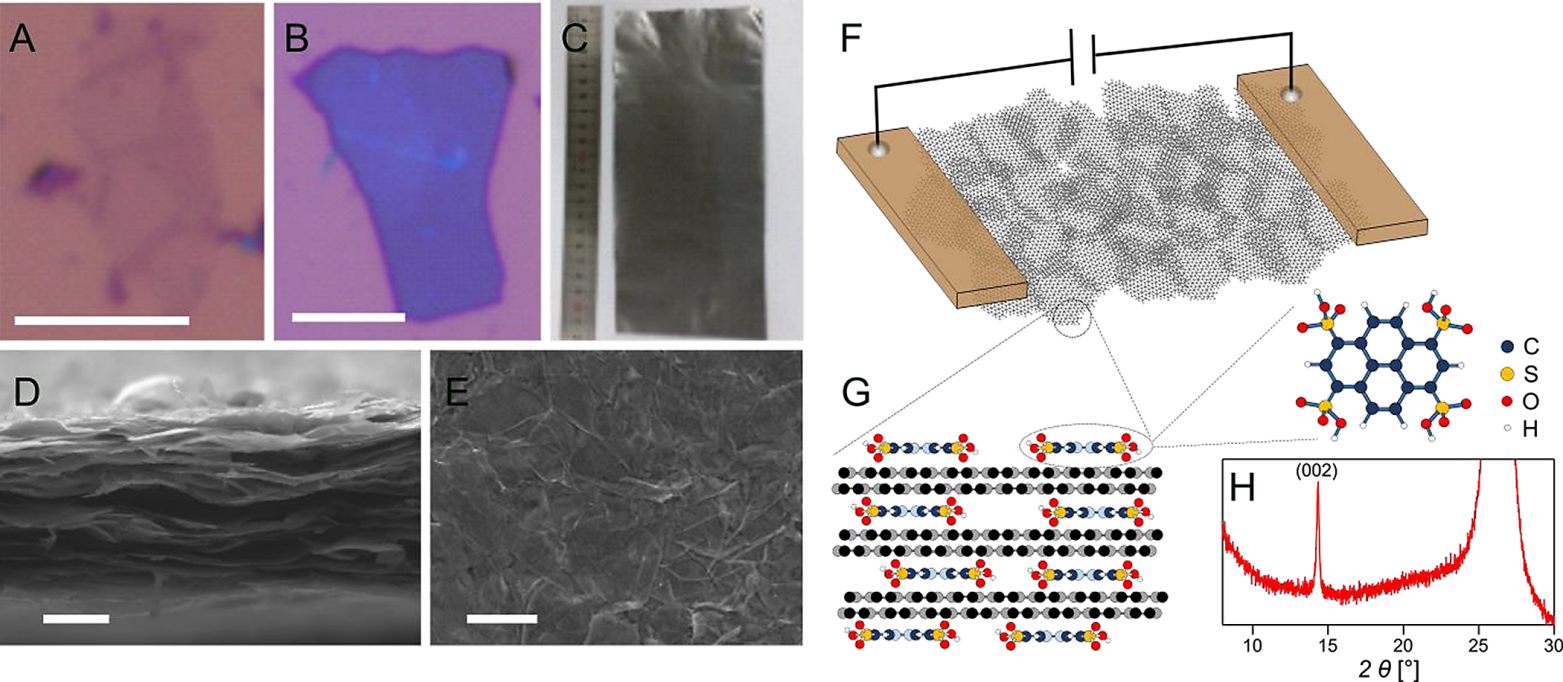
Graphene laminates made of large-area nanosheets. Panels (A) and (B) show a monolayer and 12.5 nm-thick graphene nanosheets deposited on a silicon wafer with the 290 nm oxide layer. Graphene nanosheets were assembled into a highly aligned laminate upon solution casting and mechanical compression (C), where a high degree of alignment was observed in the SEM cross-section image, Panel (D). Panel (E) shows the top view of the laminates where randomly stacked nanosheets are visible. Panel (F) schematically shows the charge transport in such a randomly stacked network. Graphene nanosheets contain an organic molecule, tetra-sulfonated pyrene, mainly in the stage-2 intercalated region, Panel (G).^24^ X-ray diffraction pattern (H) of the laminate confirms such a structure. Scale bars in (A), (B), (D), and (E) correspond to 5 μm.

All laminates discussed in this report were prepared by blade coating of a Polyethylene terephthalate (PET) film with an additive-free graphene slurry with a solid content of around 50 g·L^–1^ in *N*-Methyl-2-pyrrolidone (NMP). The slurry was obtained from the dispersion of large-area graphene nanosheets in NMP produced by chlorosulfonic acid-assisted liquid phase exfoliation.^24^ The nanosheets’ lateral size covers a range between 1 and 20 μm with thicknesses ranging from 1 to around 40 graphene layers, Figure 1A,B. The final density of the dried and calendared laminates was between 1.6 and 2.0 g·cm^–3^, depending on the quality and density of the precursor graphite crystals. The thickness of the final laminates shown in Figure 1C was in the range of 10–20 μm.

The high aspect ratio of the nanosheets and the postprocessing mechanical compression led to their alignment, which is essential for compact stacking and high flexibility.^5^ The SEM cross-section images of the laminates, Figure 1D, confirmed the high degree of in-plane alignment of graphene nanosheets along with the complete twist misorientation evident from the top view SEM imaging, Figure 1E. Although the graphene slurry formulation is completely additive-free, the individual nanosheets are intercalated with tetra-sulfonated pyrene (s-Py), which is crucial for their large aspect ratio,^24^Figure 1G. The X-ray diffraction pattern obtained from these nanosheets, Figure 1H, appears to have the signature of the stage-II intercalation phase, suggesting that nanosheets comprise several aligned graphene bilayers, and the laminate is a collection of planarly stacked randomly twisted nanosheets with different overlap areas.

The doping type of the laminates was fine-tuned through controlled thermolysis of s-Py molecules through annealing in an inert atmosphere (Ar/H_2_ or Ar) at up to 2200 °C. Here, we exploited the electron-accepting nature of s-Py, which would be a natural *p*-type dopant for the as-prepared laminates^26^ and the fact that the pristine pyrene is a weak electron donor (*n*-type dopant).^27^ That makes it possible to adjust the doping type from the “as-prepared” *p*-type laminates to *n*-type laminates by controlled removal of the sulfonated groups through high-temperature annealing.

### Linear Magnetoresistance in Laminates of Large Aspect Ratio Bilayer Graphene Nanosheets

**Figure 2:**
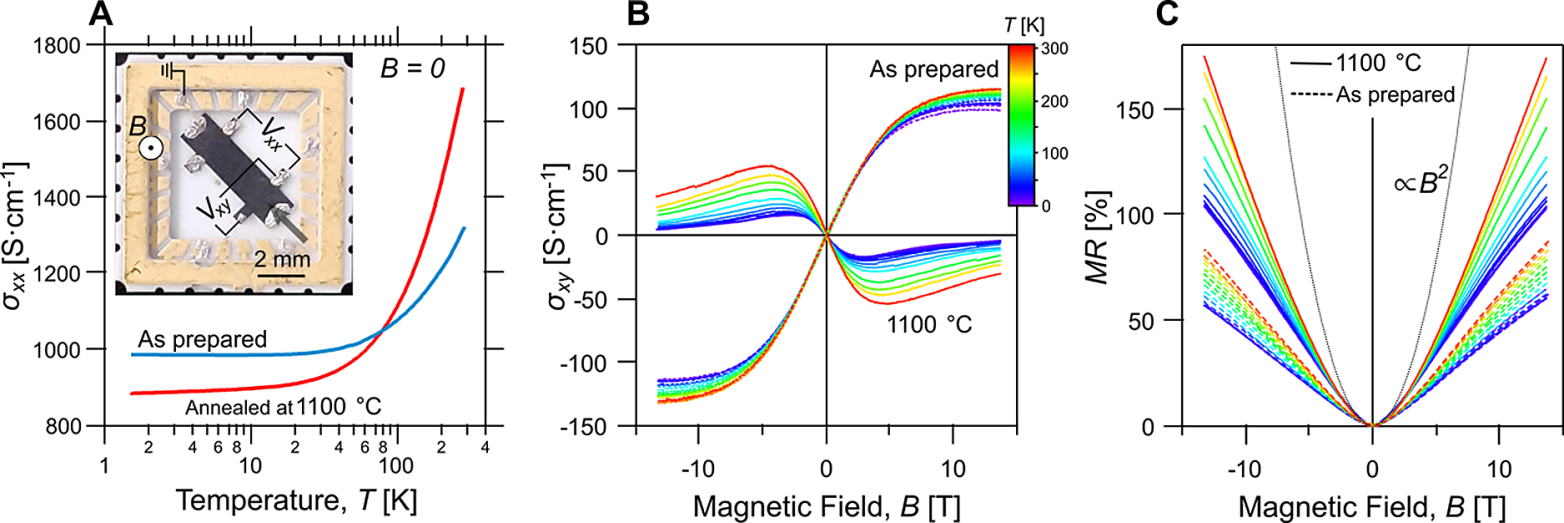
Transport characteristics of the as-prepared graphene and the laminate annealed at 1100 °C. (A) Temperature dependence of the zero-field conductivity σ_*xx*_ of the as-prepared laminate (blue line), and after annealing at 1100 °C (red line). Inset: the photograph of a typical laminate Hall bar device. (B) Hall conductivity σ_*xy*_ = −ρ_*xy*_/[ρ_*xx*_^2^ + ρ_*xy*_^2^] measured in the temperature range 1.6–300 K. (C) Magnetoresistance MR = [ρ_*xx*_(*B*)−ρ_*xx*_(*B* = 0)]/ρ_*xx*_(*B* = 0) for the as-prepared (dashed curves) and annealed at 1100 °C (solid curves) laminates measured in the same temperature as in panel (B). Quadratic response was visible in magnetoresistance at small magnetic fields (MR ∝ *B*^2^) as shown by the dotted line in panel (C), and it was used to estimate the laminate’s apparent carrier mobility as  (see main text).

The laminate samples used in magnetotransport experiments were shaped into millimeter-scale Hall bar structures by laser ablation and packaged inside standard ceramic chip carriers with the current driven in-plane and the magnetic field applied perpendicular to the laminate, Figure 2A. The measurements were performed between 1.6 K and room temperature in the presence of a magnetic field, *B*, up to 13.5 T.

The zero-field electrical conductivity, σ_*xx*_(*B* = 0) = 1/ρ_*xx*_, for the as-prepared laminate (blue trace in Figure 2A) exhibited a weak insulator-like temperature dependence with a well-pronounced saturation region below ∼20 K. The laminate annealed at 1100 °C (red curve in Figure 2A) demonstrated a similar temperature dependence with a somewhat larger conductivity change and less pronounced saturation. Note that this temperature dependence is not strong enough to be described in terms of the hopping transport as was reported earlier.^9^ We determined the room temperature carrier mobility using the measured low-field magnetoresistance, MR = [ρ_*xx*_(*B*) – ρ_*xx*_(*B* = 0)]/ρ_*xx*_(*B* = 0), as  ∼1450 cm^2^·V^–1^·s^–1^ for the as-prepared samples and ∼2100 cm^2^·V^–1^·s^–1^ for annealed samples. At the same time, the experimentally found Hall conductivity, σ_*xy*_ = −ρ_*xy*_/[ρ_*xx*_^2^ + ρ_*xy*_^2^] in Figure 2B, indicates a change from *p*-type to *n*-type of roughly the sample doping concentration.

The striking feature of the measured MR, shown in Figure 2C, is the positive linear magnetoresistance characteristic for higher magnetic fields, *B* > 3 T, observed at all temperatures and in all samples. The positive linear MR, has been observed in several materials such as graphene,^28−32^ silver chalcogenides,^33^ indium antimonide,^34^ Dirac semimetals,^35,36^ and topological semimetals.^37^ In all of those for their own peculiar reasons. Here we point out that for all our samples with different postprocessing annealing, the slope of the linear MR decreases with the decreasing temperature, which we consider as a hint toward the relevance of the phonon-mediated processes in both in inta- and interlayer transport characteristics.

**Figure 3:**
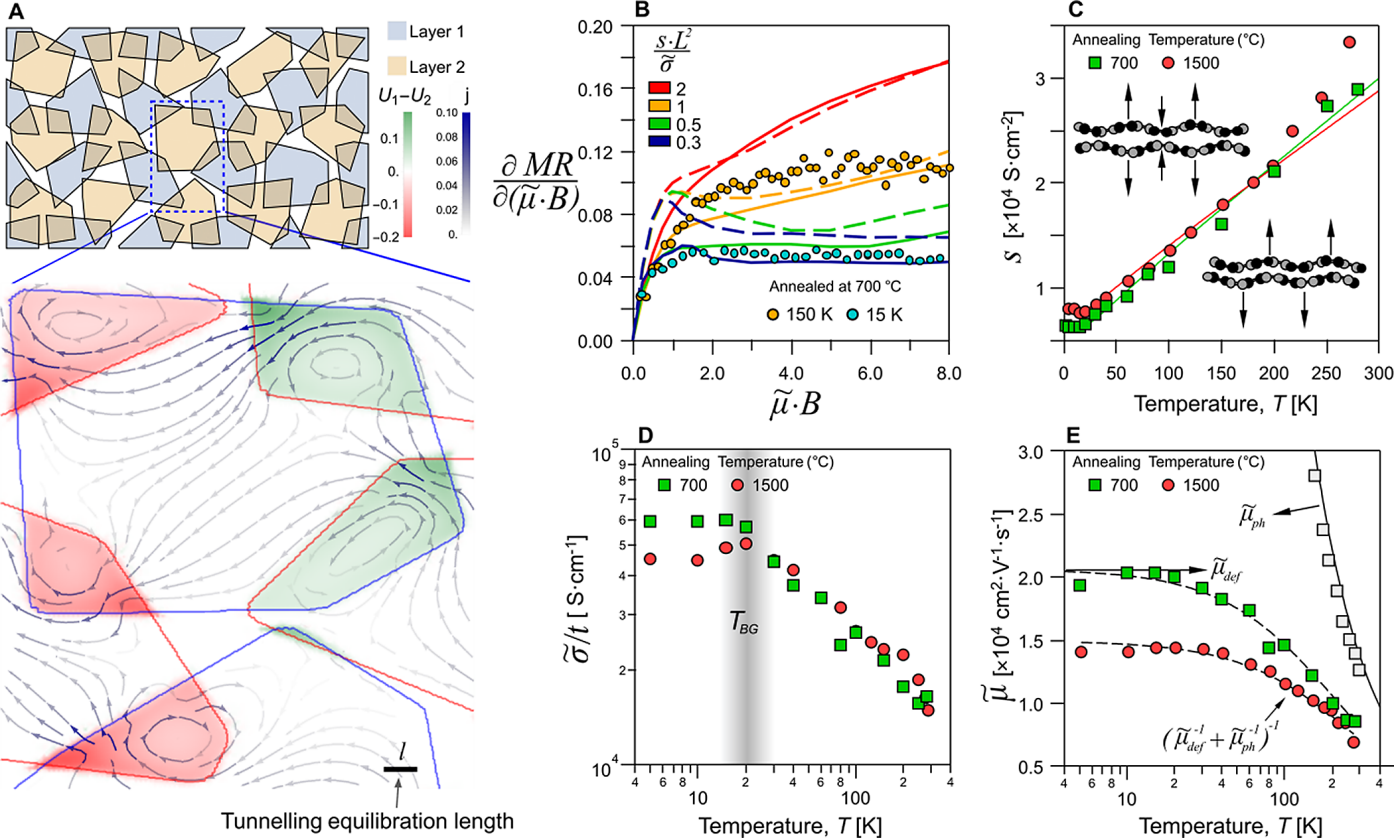
Numerical model of the linear magnetoresistance and transport parameters of individual graphene nanosheets. (A) Results of the numerical modeling illustrating the spatial distribution of the potential difference *U*_1_–*U*_2_ (color map) and total current (arrows) for two layers of polygons with the same  conductivity but opposite . Boundaries of the nanosheets are marked with blue and red curves. (B) Comparison of MR derivative for the model of overlapping random polygons (solid) and periodic array of overlapping squares (dashed). Filled circles show the experimental data 15 and 150 K from the annealed laminates extracted using the square lattice model. (C) Temperature dependence of the tunneling interfacial conductance, *s*, (symbols) extracted from the experimental data measured for two samples annealed at 700 °C (green symbols) and 1500 °C (red symbols). Solid curves show the theoretical calculations made for the phonon-assisted tunneling model, with the coupling constant g being the only fitting parameter (see text). Insets in panel (C) illustrate the graphene’s beating phonon modes considered for this calculation.^39^ (D) Individual nanosheets’ conductivity, , where *t* = 4 nm for the same two laminates. Vertical band illustrates the Bloch-Gruneisen temperature, *T*_BG_ ≈ 20 K. (E) Individual nanosheets’ carrier mobility for the laminates annealed at 700 °C (green symbols) and 1500 °C (red symbols). Dashed curves are the expected total carrier mobility defined as  with the same value of the phonon-limited lattice contribution . Gray symbols in (E) show the mobility of graphite single crystals^40,41^ limited by the same lattice contribution as in our samples.

Aiming at the quantitative analysis, we employ a model that accounts for the high intrinsic intralayer carrier mobility and weak interlayer coupling. For numerical simulations, we model a laminate as a network of overlapping nanosheets, each composed of *N*_BLG_ aligned graphene bilayers (separated by intercalant molecules), and parametrized by the lateral conductivity tensor of individual nanosheets, , (with components  =  = *en*/(1 + (μ̃*B*)^2^) and  =  = *en*^2^*B*/(1 + (μ̃*B*)^2^)) and vertical tunneling conductance per unit area of the overlapping regions, *s*, where  – is the charge carrier mobility of a nanosheet, and *n* = *N*_BLG_ρ_BLG_*E*_F_ is the carrier density per nanosheet composed of *N*_BLG_ ≈ 3.5 bilayers with the density of states ρ_BLG_ and the Fermi level *E*_F_ counted from the bilayer’s charge neutrality point. To account for sample inhomogeneity, each nanosheet is assumed to possess a single carrier type, but the position of the Fermi level (carrier concentration) varies randomly between the nanosheets. Note that in the overlap region, the interlayer tunneling makes our model mathematically equivalent to the multicarrier model used in ref (25) with the tunneling process interpreted as “carrier recombination”. While in the modeling we used various shapes of nanosheets, in Figure 3A, the typical modeling results are exemplified by an array of random overlapping polygons.

For a pair of overlapping nanosheets, denoted as “1” and “2”, the intra- and internanosheets current is related to potential distribution across the sheets as
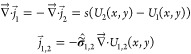
1Here *U*_1,2_(*x*, *y*) and  are the potential distribution and electrical conductivity of nanosheets 1 and 2 correspondingly. The two coupled equations in eq 1 suggest a length scale, , which characterizes the interlayer charge equilibration. We determine the overall resistivity of the film by solving numerically eq 1 for a periodic network, additionally averaging over a distribution of the Fermi energies of individual nanosheets with the mean value ⟨*E*_F_⟩, and variance δ*E*_F_ (note that in annealed samples, we have ⟨*E*_F_⟩ ≪ δ*E*_F_). An example of current and potential distribution in several overlapping nanosheets at a high magnetic field such that *B* = 10 is shown in Figure 3A.

Model in eq 1 allows us to develop an interpolation formula to describe the zero-field resistance of the laminate,
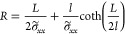
2such that  (see S.I. Section S3 for details). This model also allows us to reproduce theoretically the linear magnetoresistance shown in Figure 2C. This appears to be the result of shrinking charge equilibration length upon increasing the magnetic field to the values where μ̃*B* ≫ 1, so that , which squeezes interlayer tunneling to hotspots with the size *l*∼, Figures 3A and S1. These considerations suggest that for large enough  while at zero field *R*(0) ∝ 1/*s*, leading to
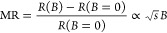
3which slope is dependent on the interlayer coupling *s* (for details, see Supporting Information, Figure S5). The linear behavior can also be argued as a network of two-terminal Hall devices.^38^ The results of numerical MR modeling are displayed in Figure 3B, where we compare a laminate built of random polygons with a periodic array of overlapping square-shaped nanosheets. In both realizations we find similar linear asymptotic MR behavior (illustrated as ∂MR/∂*B* → const), which reflects similar current density distribution and formation of the tunneling hot spots, Figure S1. Also, in Figure 3B we show two examples of the experimental data obtained from the high-temperature annealed laminates (for which ⟨*E*_F_⟩ ≪ δ*E*_F_, and *s* is used as the fitting parameter for each sample temperature). Based on this qualitative similarity, in the further data analysis we employ the square-shaped nanosheet model because of its higher numerical efficiency. While this simplified model gives only ∼30% accuracy in the absolute values of the fitting parameters, it allows the establishment of their qualitative temperature dependences, as discussed in the next section.

### Phonon-Assisted Tunneling and the Electrical Conductivity of Individual Nanosheets

The results of fitting of the experimentally observed MR – from weak magnetic fields where it is quadratic to the high-field linear regime–with  and *s* used as fitting parameters at each temperature (e.g., at 5 and 300 K; see Figure S3 for specific examples) are shown in Figure 3C–E. This gives us information about the temperature dependencies of the interlayer tunneling, Figure 3C, individual nanosheet conductivity, Figure 3D, and the intrinsic mobility of the constituent bilayers, Figure 3E.

The temperature dependence of the nanosheet mobility μ̃ (symbols in Figure 3E) is typical for a charge transport limited by defects at low temperatures and electron–phonon scattering at higher temperatures, , where  – is the defect-limited mobility found from the low-temperature saturation region and – is the temperature-dependent phonon-limited contribution. For all samples used in this study, the phonon-limited component appears to be universal and described as  = 1275/*T*^1.2^ [m^2^·V^–1^·s^–1^] (solid line in Figure 3E) – in a reasonable agreement with the values previously reported^40,41^ for bulk graphite (open squares). This phonon-limited room-temperature intrinsic carrier mobility is a sign of a high crystallinity of the constituent nanosheets and a high microscale homogeneity of the s-Py intercalant distribution.

Moreover, in all tested samples, the extracted value of the interlayer tunneling conductivity, *s*, acquires distinct linear asymptotic upon the temperature increase above 20 K, Figure 3C. We attribute this behavior to the fact that the tunneling between misaligned graphene nanosheets requires a substantial momentum transfer due to the mismatch between *K*-points in the Brillouin zones of consecutive randomly oriented bilayers. While at low temperatures this interlayer momentum mismatch can only be overcome by disorder-assisted tunneling (e.g., resonant tunneling through the s-Py orbitals), at elevated temperatures, the inelastic phonon-assisted processes kick in, compensating the momentum mismatch by the momentum transfer from or to graphene’s breathing phonon modes. Tunneling assisted by the breathing phonon modes has been previously identified as the primary transport mechanism between strongly twisted monolayer graphene flakes.^39,42^ To describe the contribution of the out-of-plane vibrations of graphene, we use the phonon-assisted tunneling conductance derived in Supporting Information, Section S4.
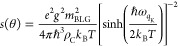
4which takes into account both spontaneous and stimulated emission/absorption of phonons. Here ρ_C_ = 7.6 × 10^–7^ kg·m^–2^ is the mass density of graphene, *m*_BLG_ ≈ 0.03 *m*_e_ is the effective mass for electrons in BLG used to estimate its density of states and ω_*q*_K__ is the energy of the beating phonon mode at momentum *q* = 2*K*sin(θ/2), connecting the *K*-points ( 17 nm^–1^) of graphenes twisted by angle θ. As shown in Figure S6, the randomness of the twist angle in the network can be accounted for by a simplified model with the same tunneling between all sheets with the twist angle fixed to θ ≈ 16°. The onset of a linear-like dependence of *s* versus *T* occurs at *T* ≈ 0.2*ℏ*ω_*q*_/*k*_B_, which suggests that both the breather and flexural modes of BLG are engaged in the tunneling conductivity and have energies at around *ℏ*ω_*q*_ ≈ 10 meV. While the temperature dependence of *T* resembles the one observed in twisted bilayers of graphene,^39^ its lower absolute value in Figure 3C can be attributed to a larger separation between the bilayers caused by the presence of s-Py molecules.

### All-Graphene Bipolar Thermocouple

**Figure 4:**
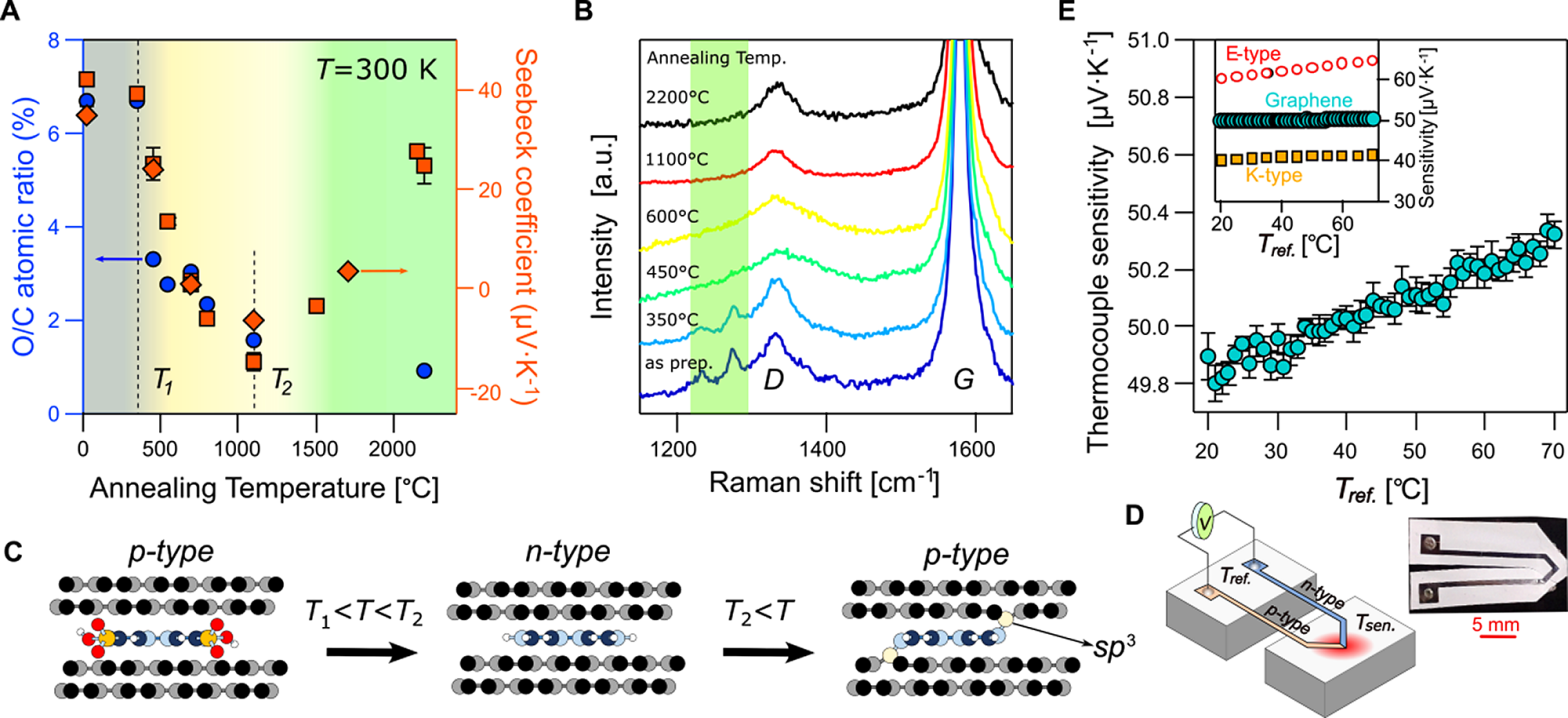
Tuning the thermopower of graphene laminates by s-Py charge transfer. (A) Correlation between the Seebeck coefficient (orange symbols) and the oxygen-to-carbon atomic ratio obtained from the XPS spectrum (blue circles) for the laminates annealed at different temperatures. Temperatures *T*_1_ ≈ 350 °C and *T*_2_ ≈ 1100 °C mark the onset of the desulfonation and dehydrogenation of an s-Py molecule. Orange diamond and square symbols denote the thermopower data obtained from the laminates derived from different graphite materials (Ashbury Graphite and Graphenium flakes, NGS). (B) Raman spectra of graphene laminates annealed at different temperatures demonstrate the disappearance of the band between 1230 and 1275 cm^–1^ (highlighted in green) associated with the s-Py molecules. (C) Schematics illustrate the thermolysis of s-Py, which is responsible for the reversal of the conductivity from *p*-type to *n*-type at low annealing temperatures (*T*_1_ < *T* < *T*_2_). At higher annealing temperatures (*T* < *T*_2_), the dehydrogenation and formation of the out-of-plane sp^3^ carbon bonds responsible for the reentrant *p*-type conductivity. (D) Schematics of the all-graphene thermocouple device are made of two graphene laminate strips with the individual Seebeck coefficients tuned by annealing. Inset: The photograph of the actual thermocouple device. (E) Thermocouple sensitivity measured at different reference temperatures, *T*_ref_ as illustrated in panel (D). Sensitivity is comparable to the commercial *K*-type and *E*-type thermocouples, as shown in the inset (NIST database, https://srdata.nist.gov/its90/main/).

As noted above, the postprocessing annealing at 1100 °C leads to the reversal of doping from *p*-type to *n*-type, evidenced by the Hall conductivity sign change (Figure 2B). To gain more information about the doping mechanism, we correlated the thermoelectric measurements with the X-ray photoelectron spectroscopy (XPS) analysis and Raman spectroscopy. Figure 4A combines the data for thermoelectric power and the XPS oxygen-to-carbon content ratio (O/C) found in laminates annealed at different temperatures. The thermopower correlates well with the O/C ratio up to around 1100 °C. Initially, both remain unchanged to *T*_1_ ≈ 350 °C and then start to decrease–the thermopower from +42 to about −14 μV·K^–1^ and the O/C ratio from 6.7 to 1.6% due to the removal of sulfur-bounded oxygen (Figure S9). The onset at *T*_1_ is attributed to the carbon–sulfur bond cleavage reaction,^43^ and signifies the desulfonation of the s-Py acceptor molecules^26^ through the thermolysis reaction. The desulfonation of s-Py causes a gradual change from the *p*-type conductivity to *n*-type when the pyrene is stripped off from the sulfonic groups and acts as a donor dopant.^27^ The loss of the sulfonic groups is also confirmed by the Raman spectroscopy (Figure 4B), showing a gradual disappearance of the bands between 1200 and 1300 cm^–1^ associated with s-Py.^44^ For the samples annealed at *T*_2_ ≈ 1100 °C and higher, the sulfur content was undetectable in XPS signal (Figure S9).

Further annealing above *T*_2_ ≈ 1100 °C returns *p*-type doping of laminates despite the saturated O/C ratio (Figures 4A and S10). We suggest that at such high temperatures, pyrene molecules undergo cleavage of their carbon–hydrogen bonds and formation of new σ-bonds with the carbon atoms from the adjacent graphene layer, inflicting some sp^3^-hybridization,^45^Figure 4C. The electron deficiency created in these new sp^3^ bonds leads to overall *p*-type doping of graphene, which explains the observed reversal of the Seebeck coefficient in material annealed at higher temperatures, Figure 4C. At the same time, the formation of the out-of-plane sp^3^ bonds produces point-like defects, which show up in a ∼30% increase in the Raman D peak intensity. The introduction of additional point-like defects also leads to a proportional drop in carrier mobility, as seen in Figure 3E.

All these give access to the controlled tuning of laminates’ Seebeck coefficient by the postprocessing annealing. The thermopower values and ability to vary its sign we achieve in laminates, e.g., as prepared and annealed at 1100 °C, are comparable to those of commonly used metal thermocouple alloys. To demonstrate the potential application of such laminates, we fabricated a proof-of-principle all-graphene thermocouple made of combined *p*- and *n*-type laminates, Figure 4D, as-prepared and annealed at 1100 °C, respectively. The fabricated thermocouple performs with a sensitivity of ∼50 μV·K^–1^, Figure 4E. With reference to the second reversal of the doping type at higher annealing temperatures, the result in Figure 4A suggests that one can make an equally sensitive thermocouple using the 1100 and 2200 °C annealed laminates which would be safe to operate up to 1000 °C.

## Conclusions

This work showed that the graphene laminates prepared by the acid-assisted liquid-phase exfoliation technique followed by a high-temperature annealing result in high electrical conductivity characterized by a linear MR regime in a strong magnetic field (*μB* ≫ 1). The linear MR regime is explained by a combination of tunneling coupling between the nanosheets and strong local Hall currents, both of which contribute to the total conductivity of the nanosheet network. Equipped with the MR data and numerical modeling, we disentangle the contribution of the intraplane and interplane conductivities. The observed temperature dependences of both quantities point to the electron–phonon coupling dominated transport at a temperature above 100 K, displaying the high intrinsic quality of the constituent nanosheets. We also demonstrated the ability of the described laminates to change their doping, both value and polarity, depending on the postprocessing annealing temperature, which we used to produce a proof-of-principle all-graphene thermocouple with competitive sensitivity.

## Methods

### Laminate Production

Dispersions of graphene in *N*-Methyl-2-pyrrolidone (NMP) were obtained through a chlorosulfonic acid-assisted exfoliation method, as previously described in our work.^24^ Our analysis revealed that the median size and thickness of the nanosheets were 5.6 μm and 4.0 nm, respectively.^26^ The dispersions were centrifugated at 12,000 rpm for 1 h to separate the nanosheets from the acid and excess pyrene. The resulting sediments were collected and redispersed in NMP to form a homogeneous slurry containing 5 wt % of graphene.

This viscous slurry was then directly cast onto a plastic (PET) substrate using the doctor blade coating technique without adding any other substances. The laminates were dried at 90 °C for 2 days. Since NMP has a high boiling temperature, the remaining solvent was removed by immersing the dried film in a hot water bath. The water removed most of the residual NMP, and the film was subsequently dried at 90 °C overnight. To further increase the film’s density, the samples were mechanically compressed using a twin roller press and then manually peeled off from the substrate.

Subsequently, the samples were annealed in an electric furnace (Carbolite quartz tube furnace) under an Ar/H_2_ atmosphere at the target temperature for 2 h, with a heating and cooling rate of 2 °C·min^–1^. For samples annealed at temperatures above 1100 °C, a different furnace (FCT Systeme GmbH) was used in an Ar atmosphere, maintaining the same annealing rate and duration. After annealing, all samples underwent calendering in a hydraulic press at 350 bar.

### Material Characterization

Scanning electron microscopy (Tescan Mira 3 FEG-SEM) or optical microscopy (Nikon Eclipse LV100ND) was employed to determine the samples’ thickness.

The elemental composition of samples was evaluated using XPS on an ESCA2SR spectrometer (Scienta Omicron GmbH) or HAPEX (HAXPES-Lab, Scienta Omicron GmbH). Raman spectra were captured on Renishaw inVia using a 633 nm laser. X-ray diffraction patterns were collected on a Rigaku SmartLab diffractometer using Cu K-α radiation.

### Transport Measurements

The samples were cut into Hall bar geometry, 1.5 mm × 3.0 mm (between the potential probes), using the laser photoablation system (M-Solv MSV-100) and then bonded inside a ceramic chip carrier (see inset of Figure 2A). The transport measurements were performed in a cryogen-free variable temperature cryostat equipped with a 14 T superconducting magnet (Cryogenic Limited, CFMS-14T). To determine the longitudinal, ρ_*xx*_, and transversal, ρ_*xy*_, resistivities, the longitudinal, *V*_*xx*_, and Hall, *V*_*xy*_ voltages were measured by applying a current of *I* = 10 μA using a lock-in amplifier (Stanford Research Systems SR860). The bulk conductivity tensor was then calculated from the geometry and thickness of the samples.

### Thermoelectric Measurements

The Seebeck coefficient of the samples was measured on a home-built setup at room temperature. In brief, the samples were mounted between two aluminum posts with individual PID temperature control achieved with miniature Peltier modules (European Thermodynamics, APH-031-10-13-S) and a pair of high-precision platinum RTD (Innovative Sensor Technology, P0K1.232.4W.K.010). The dual-channel source meter units (Keithley 2636B) operated with LabView software provided the temperature reading and heating power supply, and the low-noise nanovoltmeter (Keithley 2182A) was used to measure the thermoelectric voltage.

## Supporting Information Available

Detailed description of the conductivity numerical model, a discussion of the nanosheet electrical properties obtained using this model, an expanded discussion of the origins of the linear magnetotransport in a graphene laminate, a detailed theoretical description of the phonon-assisted tunneling processes, an assessment of the number of the point defects in individual nanosheets using the Raman spectrometry, the XPS compositional analysis of the laminates used in this study, an example of weak localization observed for some of the samples, and comparison of the graphene thermocouple performance with the available literature data (PDF)

## Accession Codes

The Matematica code (v.12 and v.13 compatible) was used to fit the experimental magnetoresistance data with the numerical model described in the main text. Code is available at: https://github.com/slizovskiy/GrapheneLaminate/releases/tag/supplementary.
