# Supplementary material for: Ultimate Charge Transport Regimes in Doping-Controlled Graphene Laminates: Phonon-Assisted Processes Revealed by the Linear Magnetoresistance
Source: ACS Nano. 2024 Aug 8;18(33):22172–80. doi: 10.1021/acsnano.4c05512 (PMC11342362; doi:10.1021/acsnano.4c05512)
Supplement: Supplementary file 1 — nn4c05512_si_001.pdf [file nn4c05512_si_001.pdf]

Supporting Information

Ultimate charge transport regimes in doping-controlled graphene laminates: phonon-assisted processes revealed by the linear magnetoresistance.

Mohsen Moazzami Gudarzi [1], Sergey Slizovskiy [1], Boyang Mao [1,3], Endre Tovari [4], Gergo Pinter [2], David Sanderson [2], Maryana Asaad [2], Ying Xiang [2], Zhiyuan Wang [2], Jianqiang Guo[2], Ben F. Spencer [2], Alexandra Geim [5], Vladimir I. Fal'ko [1,6,7] and Andrey V. Kretinin[,1,2,6]*

[*] E-mail: andrey.kretinin@manchester.ac.uk

[1] Department of Physics and Astronomy, The University of Manchester, Oxford Road, Manchester, M13 9PL, U.K.

[2] Department of Materials, The University of Manchester, Oxford Road, Manchester, M13 9PL, U.K.

[3] Cambridge Graphene Centre, Department of Engineering, University of Cambridge, 9 JJ Thomson Ave, Cambridge CB3 0FA, U.K.

[4] Department of Physics, Institute of Physics, Budapest University of Technology and Economics, Műegyetem rkp. 3, H-1111 Budapest, Hungary

[5] Department of Physics, Harvard University, Cambridge, Massachusetts 02138, United States

[6] National Graphene Institute, The University of Manchester, Oxford Road, Manchester, M13 9PL, U.K.

[7] Henry Royce Institute for Advanced Materials, The University of Manchester, Oxford Road, Manchester, M13 9PL, U.K.

S1. Numerical model for conductivity in laminate

As discussed in the main text and illustrated in Figure 1G, the laminate is made of few-layer graphene nanosheets, which are intercalated with pyrene molecules at every second layer (stage II intercalation), effectively splitting the multilayer flakes into a collection of aligned bilayers. The electrical conductivity between the aligned bilayers is good since they are aligned, while the conductivity between different misaligned flakes is incoherent and is governed by impurity-assisted and phonon-assisted tunneling mechanisms. Experimental data indicate a weak dependence of resistivity on the annealing temperature when the Hall resistance changes its sign. Such behavior indicates an ambipolar conduction mechanism involving carriers with a different sign of electric charge. However, the bilayer graphene has only one carrier type at a specified Fermi level: either electrons or holes. Coupling between the bilayers can induce ambipolar conduction, but this effect would be absent in the thinner flakes and would have to originate from the hopping process to the next nearest layer, which has a typical energy of $\gamma_2 \approx 20$ meV in graphite, and we expect a much smaller value in intercalated flakes due to the larger vertical separation between bilayers. An alternative approach is to assume that all the flakes have an electronic dispersion of several weakly coupled graphene bilayers, but different flakes have different Fermi levels (relative to the charge-neutrality point). To further simplify the model, we assume that each flake consists of

$N_{\mathrm{BLG}} \approx 3.5$ bilayers (corresponding to the average flake thickness found in the experiment) share the same Fermi level. The Fermi level positions are assumed to be normally distributed across the set of flakes with dispersion δE_{F} and expectation value $\langle E_{\mathrm{F}} \rangle$. The flakes are chosen to be of a square $L \times L$ ($L = 0.8$ in the numerical model) shape and tile two layers with $0.2L$ gaps, see Figure S1A. In the regions where flakes overlap, there is a vertical tunneling conductivity per unit area s.

We have also verified that the model utilizing random polygons, Figure S1B, produces qualitatively similar results despite notably smaller overlap areas.

The linear magnetoresistance (MR) in our model appears from overlapping flakes being connected in a two-terminal configuration, and the MR is only weakly sensitive to the relative sign of the Hall coefficient of overlapping flakes. In Figure S1, we assumed an opposite (*p*- and *n*-) doping of the overlapping flakes, and in Figure S2 we compare the cases of the opposite and the same doping, showing that both arrangements result in almost the same MR response.

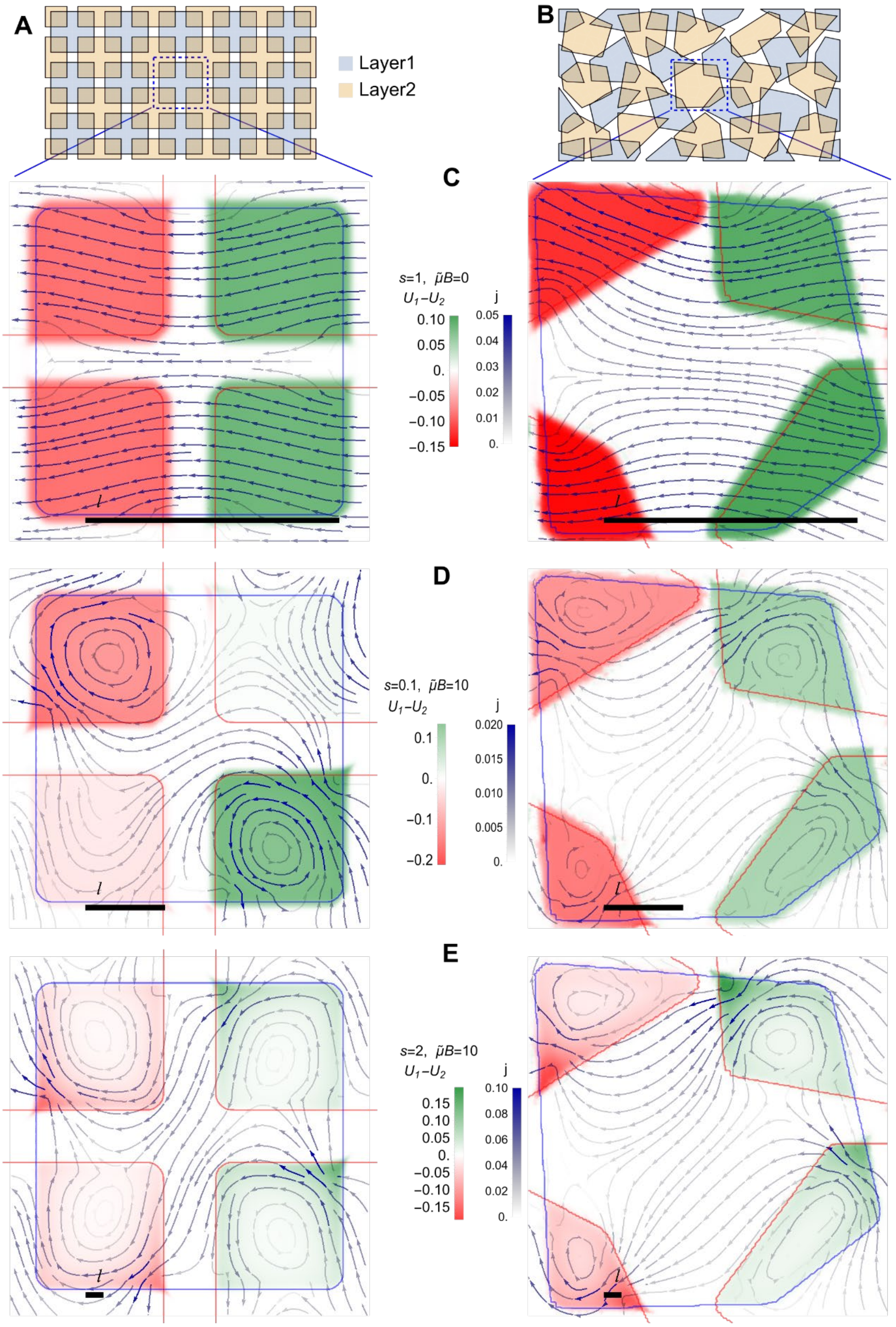

Figure S1. Panels A and B: Nanosheet network geometry of flakes used in numerical modeling. Results for two layers of oppositely-doped squares (left) and polygons (right) are

shown. Panels C, D and E: examples of total current distribution (arrows) and the potential difference between the layers (red-to-green color scale) for $B = 0$ (C) and $\tilde{\mu}B = 10$ (D and E). The black scale bar corresponds to tunneling equilibration length $l = \sqrt{\frac{en\tilde{\mu}}{2s(1+(\tilde{\mu}B)^2)}}$, which is comparable to flake size in (C, D) and notably shorter in (E). Dimensionless tunneling s corresponds to s_{model} as given in the text. Overlapping flakes are assumed to have opposite (p- and n-) doping.

The flake carrier mobilities are chosen as $\tilde{\mu}$ for electrons and 0.9 $\tilde{\mu}$ for holes (when $E_{\text{F}} < 0$) to account for a larger effective mass of holes in bilayer graphene and graphite.[1] We use the Drude formula for in-plane flake conductivity $\tilde{\sigma}_{xx} + i\,\tilde{\sigma}_{xy} = \frac{\sigma_0}{1+i\tilde{\mu}B}$ where $\sigma_0 = N_{\text{BLG}}\rho_{\text{BLG}}e\,\tilde{\mu}\,E_{\text{F}}$ is determined by the Fermi level that is randomly chosen for each flake from the normal distribution. The physical vertical conductivity can be represented in the form of a dimensionless parameter

$$s_{\text{model}} = \frac{s\,L^2}{\tilde{\sigma}_{\text{typical}}}, where\ \ \tilde{\sigma}_{\text{typical}} = N_{\text{BLG}}\rho_{\text{BLG}}e\,\tilde{\mu}\,\delta E_{\text{F}}$$

is the value of in-plane conductivity determined by the typical width, δE_{F}, of Fermi level distribution.

For the two-layer model system we solve the current conservation equations numerically to find a potential distribution, $U_{1,2}$, where the current flux between the flakes is proportional to the voltage difference:

$$\begin{aligned} \vec{\nabla}\cdot\vec{j}_1 = -\vec{\nabla}\cdot\vec{j}_2 = s_{1-2}\big(U_2(x,y) - U_1(x,y)\big) \\ \vec{j}_{1,2} = -\tilde{\sigma}_{1,2}\vec{\nabla}\cdot U_{1,2}(x,y) \end{aligned} \qquad \text{(S1)}$$

magnetoresistance $MR = \frac{\rho_{xx}(B)-\rho_{xx}(0)}{\rho_{xx}(0)}$ and normalized Hall conductivity $\frac{\sigma_{xy}(B)}{\sigma_{xx}(B=0)}$ are independent of the absolute values of conductivity and flake size.

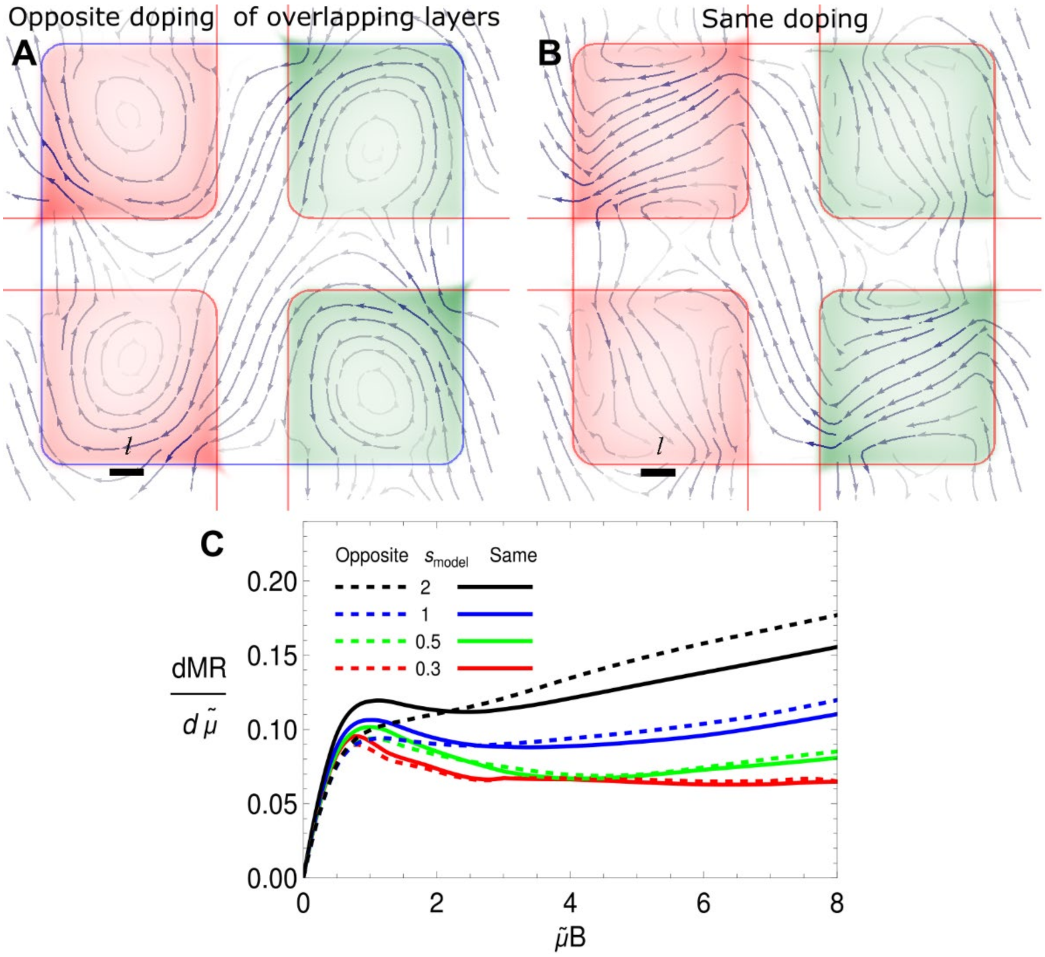

Figure S2. Panels A and B: Comparison of current distribution between the opposite (A) and the same (B) type of carriers in the two nanosheets. Plots are made for s_{model}=1 and $\tilde{\mu}\,B=10$. Panel C: Comparison of the slope of MR between the same/opposite doping combinations.

Hence, in the modeling we can choose the units where $N_{\mathrm{BLG}}\rho_{\mathrm{BLG}}e\,\tilde{\mu}\,\delta E_{\mathrm{F}}=1$ so that we are left with only two fitting parameters, s_{model} and $\langle E_{\mathrm{F}}\rangle$, and the dimensionless magnetic field, $\tilde{\mu}\,B$, as a variable. The numerical data for resistance and Hall resistance is taken in the "Hall bar" setup, where we step away from the source and drain contacts to record the voltage. The results for the model consisting of 24 square flakes are further averaged over 15 random realizations of flake Fermi levels.

To aid the numerical stability, the conductivities are further smoothened with a Gaussian kernel, as can be observed in Figure S1, and a small conductivity $10^{-5}\,\sigma_0$ is introduced in the gaps between the flakes.

Finding the parabolic magnetoresistance at low magnetic fields, we can relate the flake mobility, $\tilde{\mu}$, to the effective network "magnetic" mobility, μ, which can be extracted from the experiment, Figure S3.

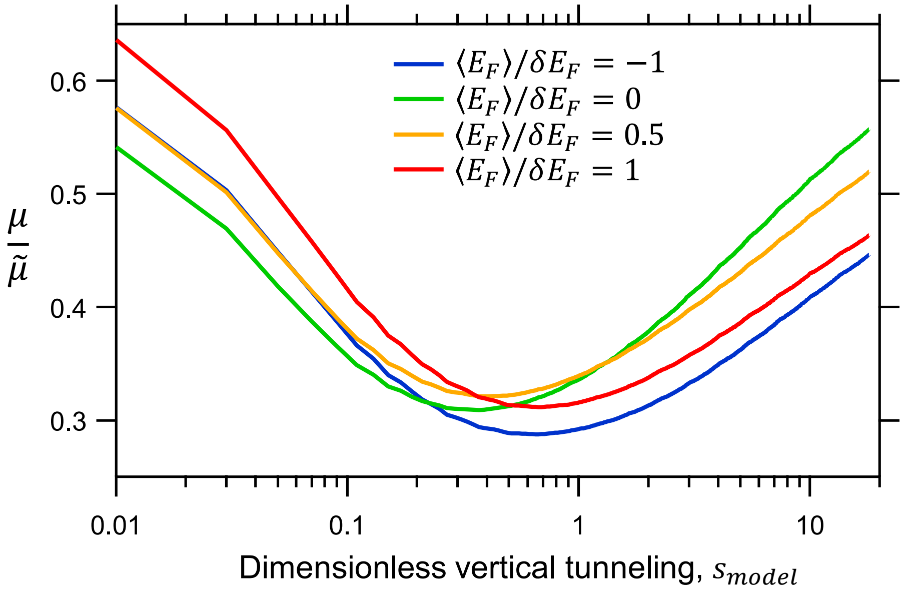

Figure S3. The ratio of network mobility to electron mobility of individual flakes, plotted versus dimensionless vertical tunneling.

Finally, we can fit the experimental data for MR and Hall resistance with simulation, finding the best-fit values for s_{model} and $\langle E_{\text{F}}\rangle$ for each measured sample at any fixed temperature. Figure 3C of the main text shows examples of the model application. We have also observed that the MR started to show saturation at very large magnetic fields, deviating from the linear trend. This effect appeared in the numerical simulation due to a large but finite resistance in the area between the flakes in the same plane, resulting in the in-plane leakage, which becomes noticeable when the flake resistance is large in high magnetic fields. We suggest

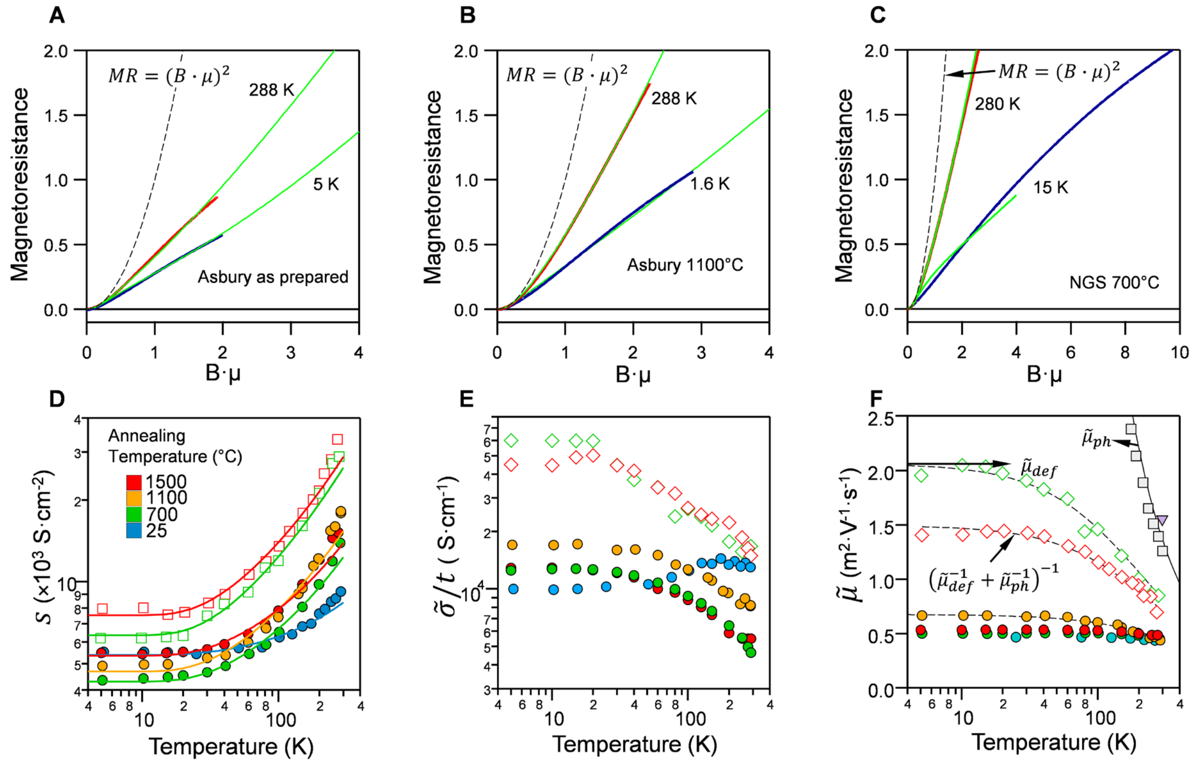

Figure S4. Panels A)-C) show the comparison of the numerical model (green lines) with experimental MR data measured at high (red lines) and low temperature (blue lines). The dashed lines show the quadratic dependence of MR on the magnetic field. Note that the magnetic field is normalized to low mobility. Data for three representative samples are shown: (A) as-prepared laminates from graphite supplied by Ashbury and two annealed samples from (B) Ashbury and (C) NGS graphite. The quality of fit for all samples and temperatures was similar. Panels D)-F) show the temperature dependence of the tunneling interfacial conductance, s in (D), the individual nanosheets' conductivity, $\tilde{\sigma}$, where $t = 4$ nm in (E) and the carrier mobility in (F) for the as-prepared laminates (blue symbols) and the laminates annealed at 700 °C (green symbols), 1100 °C (orange symbols) and 1500 °C (red symbols). Solid curves in panel D) show the theoretical calculations made for the phonon-assisted tunneling model, with the coupling constant g being the only fitting parameter (see Table S1). Filled and empty symbols represent the data for samples produced from Ashbury and NGS graphites. See the main text, Figure 3, for more details.

that a similar mechanism of in-plane leakage explains the saturation seen in the experimental data for high-quality NGS samples, Figure S4.

As expected, the results of the fitting show that all the annealed samples are strongly inhomogeneous, having an average doping smaller than doping fluctuations, $\langle E_{\mathrm{F}} \rangle \ll \delta E_{\mathrm{F}}$, while the as-prepared sample is significantly p-doped, $\langle E_{\mathrm{F}} \rangle \approx -\delta E_{\mathrm{F}}$, see Table S1.

S2. Determination of microscopic material properties with the help of the numerical model.

We use the data on average nanosheet dimensions to relate the numerical modeling results. Since the nanosheets are all different in dimensions and shape, we can only discuss the "*typical*" average values, and the results can be trusted to some numerical prefactor of the order unity. This is valuable for understanding the orders of magnitude and the temperature dependences of in-plane and out-of-plane conductivities. The typical flake conductivity,

$$\tilde{\sigma}_{xx\,\mathrm{flake}} \approx N_{\mathrm{BLG}} \rho_{\mathrm{BLG}} e\, \tilde{\mu}\, \delta E_{\mathrm{F}} \approx \frac{\sigma_{xx\,\mathrm{phys}}\, t}{\sigma_{xx\,\mathrm{model}}} \qquad \text{(S2)}$$

is determined by comparing the modeled network conductivity to the experimentally measured one, where we use the average flake thickness $t \approx$ 4 nm[2]. With the help of the density of states in the graphene bilayer $\rho_{\mathrm{BLG}} \approx \frac{\gamma_1}{\pi \hbar^2 v_{\mathrm{F}}^2}$ (with $v_{\mathrm{F}} = 10^6$ m s^{-1}, γ_1=0.35 eV) and average number of bilayers per flake, N_{BLG}=3.5, we may estimate the magnitude of the spread of Fermi levels, δE_{F}. The results are presented in Table S1.

Table S1: Sample parameters estimated from fitting the experimental data with modeling results. The parameters are: δE_{F} – variance of Fermi level distribution; δn – variance of the charge carrier density; $\langle E_{\mathrm{F}} \rangle$ – average Fermi level of the flakes; $\langle n_e \rangle$ – average charge carrier density; $\sigma_{zz,T=0}$ – temperature-independent part of vertical conductivity, ascribed to impurity-

assisted tunneling; g – coupling constant of electrons to beating mode of phonons, responsible for temperature-dependent part of vertical conductivity S – Seebeck coefficient measured at 300 K.

Material/Annealing	δE_F (meV)	δn (10^{18} cm^{-3})	$\langle E_F \rangle$ (meV)	$\langle n_e \rangle$ (10^{18} cm^{-3})	$\tilde{\sigma}_{zz,T=0}$ (S m^{-1})	g (eV Å^{-1})	S (μV K^{-1})
Ashbury, as-prepared	80	18	-60	-13	0.21	0.034	42
Ashbury, 700°C	30	7	-1	-1	0.17	0.055	1.5
Ashbury, 1100°C	55	12	8	8	0.19	0.063	-14.6
Ashbury, 1500°C	30	7	-2	-2	0.21	0.056	-3.4
NGS, 700°C	29	6	-1	-1	0.27	0.086	0.9
NGS, 1500°C	70	16	-3	-3	0.30	0.089	-

Having found the dimensionless vertical conductivity, s, we use the knowledge of the average flake size, $L \approx 5.6$ nm, to estimate the vertical conductivity of randomly oriented flakes,

$$\sigma_{zz} = s\,t = \frac{s_{\mathrm{model}}\, t\, N_{\mathrm{BLG}} \rho_{\mathrm{BLG}} e\, \tilde{\mu}\, \delta E_{\mathrm{F}}}{L^2} = s_{model}\, \frac{t^2}{L^2} \frac{\sigma_{xx\,\mathrm{phys}}}{\sigma_{xx\,\mathrm{model}}} \quad \text{(S3)}$$

(note that here $\sigma_{xx\,model}$ is dimensionless since it is measured in the units of $N_{\mathrm{BLG}} \rho_{\mathrm{BLG}} e\, \tilde{\mu}\, \delta E_{\mathrm{F}}$).

S3. Origins of the linear magnetotransport in graphene laminate

Here we present the physical picture underpinning our numerical results. Before proceeding to magnetoresistance, let us switch off the magnetic field.

To gain understanding, we consider a 1D model of two overlapping flakes with conductivities σ and lengths L. We are looking at the current transport from point $x = 0$ on flake 1 to point $x = L$ on flake 2. The current conservation equations in the region $[0, L]$ are reduced to

$$j_{1,2} = -\sigma U'_{1,2}\,;\; j_1' = -j_2' = s\,(U_2 - U_1) \qquad \text{(S4)}$$

with boundary conditions $j_1(L) = j_2(0) = 0$.

The solution features a "*carrier recombination length*", $l = \sqrt{\frac{\sigma}{2\,s}}$ in the exponent, and the voltage difference between the flakes grows as $U^1 - U^2 \sim e^{\frac{x}{l}}$ near the edge. So, when $L \gg l$, the tunneling happens mainly in the region of width l near the edges of the nanosheet overlap region, while in the opposite limit, when $L \ll l$, the tunneling happens homogeneously through all of the overlap region. The resistance (of a unit length in the "y"-direction along the overlap) between points $x = 0$ on flake 1 and $x = L$ on flake 2 behaves as

$$R = \frac{L}{2\,\sigma} + \frac{l}{\sigma}\coth\frac{L}{2\,l} \approx \begin{cases} 1/(L\,s)\,, & l \gg L \\ L/(2\,\sigma) + 1/\sqrt{2\,\sigma\,s}, & l \ll L \end{cases} \qquad \text{(S5)}$$

The first term corresponds to the in-plane resistance, and the second term mostly comes from the tunneling between the flakes. In the weak tunneling regime, $l \gg L$, the second term dominates, and the resistance saturates to $R \approx 1/(L\,s)$, where it corresponds to tunneling being homogeneously distributed over the flake overlap area, in the opposite limit of strong tunneling, $l \ll L$, the tunneling happens only in the region of width l around the boundary of flake overlap region and the resistance behaves as $R \approx L/(2\,\sigma) + 1/\sqrt{2\,\sigma\,s}$. In this regime, the flakes have the same voltage distribution deep inside the overlap region and work together exactly like in a standard two-carrier model. Note that in a wide intermediate range, the $1/\sqrt{s_{\mathrm{model}}}$ term dominates over a constant term.

The magnetoresistance of laminates has two basic regimes: the standard quadratic MR at a low magnetic field, which is very similar but slightly weaker than the MR of a single flake,

and a linear regime at a high magnetic field, $\tilde{\mu}B \gg 1$. In this latter regime, the tunneling between the flakes happens mainly along the edges and corners of domains formed by flake intersections, Figure 3B. To understand this behavior, we note that it is the $\tilde{\sigma}_{xx} = \frac{\sigma_0}{1+(\tilde{\mu}B)^2}$ component of conductance that is relevant to the 1D model discussed above, while $\tilde{\sigma}_{xy}$ component describes the current along the edge. Plugging $\sigma = \tilde{\sigma}_{xx}$, we see that with a growing magnetic field, we are inevitably approaching the strong tunneling regime, $l \sim 1/B \ll L$, so the tunneling current between the flakes gets concentrated in the narrow strips of width l along the edge, leading to the linear MR as described in the main text. Here, we also mention that linear MR regime can be achieved even at not-so-high magnetic fields and not-so-weak vertical tunneling, where we only need that $\tilde{\mu}\, B > 1$, but we can still have $l \sim L$. In this regime, the resistance is determined by the two-terminal magnetoresistance of the flakes, connected via overlapping parts, $R \propto \frac{1}{\sigma_{xy}} \propto \frac{1}{B}$. Since the resistance at zero magnetic fields behaves approximately as $R(B=0) \propto 1/\sqrt{s}$ in a wide realistic range of s, we get for the magnetoresistance:

$$MR(B,s) = \frac{R(B) - R(B=0)}{R(B=0)} \propto B\sqrt{s}, \qquad \text{(S6)}$$

as shown in Figure S5.

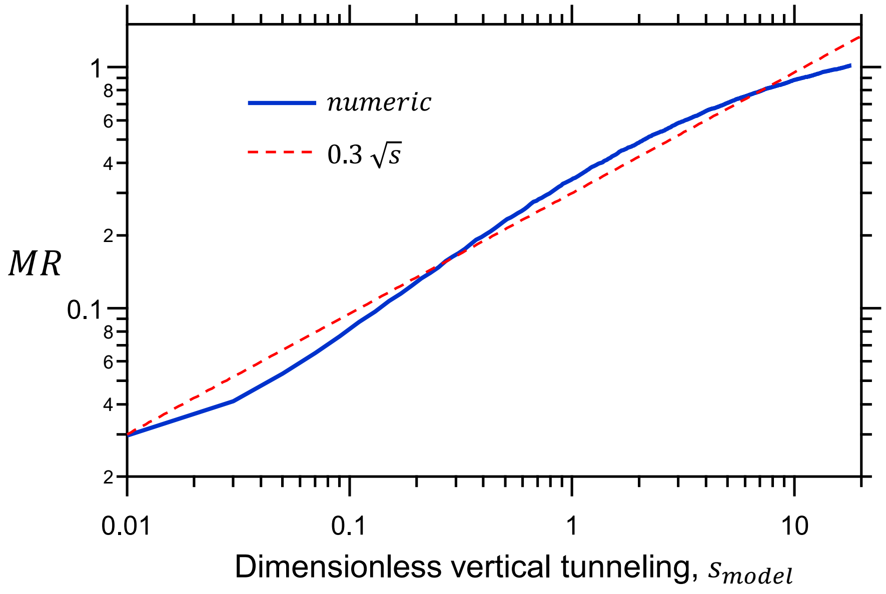

Figure S5. The slope of linear MR as a function of vertical tunneling conductance.

S4. Phonon-assisted tunnelling

This section presents a modified version of considerations in[3] for phonon-assisted tunneling between the flakes. Consider the process of tunneling between layers, assisted by the emission/absorption of the phonons with momentum $q_K \approx 2\,K \sin\theta/2$, connecting the two twisted Dirac cones, where $K = 4\frac{\pi}{3\,a} \approx 17\ \mathrm{nm}^{-1}$. The main contribution comes from the flexural breathing modes resulting in the distance beating between adjacent layers[3].

The phonon-mediated current due to bias V can be expressed as

$$I = \frac{4\,\pi\,e}{\hbar}\sum_{\{k,k'\}} M_{k'}^{k} f(E_k)\big(1 - f(E_{k'} + e\,V)\big) - M_k^{k'} f(E_{k'} + e\,V)\big(1 - f(E_k)\big) \quad \text{(S7)}$$

and

$$M_{k'}^{k} = |\langle \psi_k \mid H_{e-ph}\, \psi_{k'}\ \rangle|^2\ \left[n_q\, \delta\big(E_{k'} - E_k - \hbar\omega_q\big) + \big(1 + n_q\big)\delta\big(E_{k'} - E_k + \hbar\omega_{-q}\big)\right] \quad \text{(S8)}$$

The electron-phonon matrix element may be approximated as

$$|\langle \psi_k \mid H_{e-ph} | \, \psi_{k'}\ \rangle|^2 \approx \frac{1}{4}\frac{g^2\hbar}{\rho_C\,\omega(q_K)} \quad \text{(S9)}$$

where g – is an electron-phonon coupling constant arising due to modulation of inter-layer hopping of electrons ($g \approx 0.34$ eV Å^{-1} was suggested in[3] for pure twisted graphene, while we expect smaller values due to higher inter-plane distance in stage II intercalated flakes); here ρ_C is the mass density of graphene and the factor ¼ accounts for a probability of an electron in the bilayer to be at the right layer for the tunneling.

Assuming infinitesimal bias V, we then obtain the vertical conductivity

$$s(\theta) = \frac{e^2 g^2 m_{\mathrm{BLG}}^2}{4\,\pi \hbar^3 \rho_{\mathrm{C}} k_{\mathrm{B}} T}\left[\sinh\left(\frac{\hbar\omega_{q_{\mathrm{K}}}}{2\,k_{\mathrm{B}}T}\right)\right]^{-2} \tag{S10}$$

The phonon frequency, ω_q, can be estimated via the phonon energy near the *A*-point of graphite as $\omega_q \approx \sqrt{\omega_0^2 + \kappa\, q^4/\rho_C}$ where we expect that the phonon gap is smaller than the graphite value $\hbar\omega_0 < 8.9$ meV (due to larger separation between bilayers) and the bending rigidity $\kappa = 1.65$ eV.[4] We choose $\hbar\omega_0 = 5$ meV, but the result weakly depends on this choice due to dominance of the bending rigidity term for all not-so-small twist angles. A possible internal strain of the flakes would change the phonon dispersion as $\omega_q \approx \sqrt{\omega_0^2 + \kappa \frac{q^4}{\rho_C} + u_{xx} v_{\mathrm{L}}^2\, q^2}$, where u_{xx} is strain and v_{L} is a speed of sound,[5] which leads to a negligible change for any realistic strain values. As the temperature exceeds 200 K, we see an additional contribution to effective tunneling. It might be arising either from the excitation of higher-energy phonon branches[3] leading to an increase of conductivity between the nanosheets or due to increased in-plane conductivity through possible in-plane *p-n* barriers in the flakes, which were not taken into account in our modeling. We do not add this contribution to avoid extra fitting parameters.

To account for the random orientation of flakes in the laminates, we need to map the random twist angles conductivity model to our simpler model, which assumes the same tunneling conductivity between all the flakes. For this, we construct a large 50×50 square lattice

electrical network with conductance of each link set according to equation (S1) with a randomly drawn twist angle. Then, we find an effective mean conductance s_{eff} in such a way that the same square lattice but with conductance s_{eff} of each link gives the same network conductivity. It turns out that s_{eff} is rather close to the result for $\theta = 16°$, as shown in Figure S6. We note that this result differs from what one gets by simply averaging $s(\theta)$ over the twist angle.

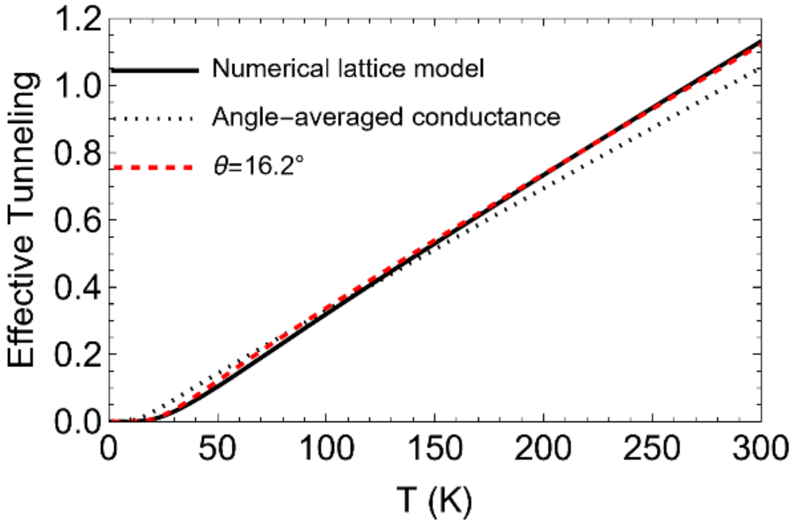

Figure S6. Effect of the twist angle of nanosheets on the network conductivity. The conductivity of the random-twist network is well approximated by the conductivity of the network with a fixed twist angle of around 16°.

The result for σ_{zz} extracted from comparing the modeling with the experiment is then fitted as $\sigma_{zz} = \sigma_{zz\,impurity} + t\, s_{\text{eff}}$, where $s_{\text{eff}} \approx s(16°)$

a sum of a temperature-independent value from impurity-assisted tunneling and a phonon-assisted contribution. Here $t \approx 4$ nm is an average thickness of flakes, and the electron-phonon coupling g is treated as a fitting parameter. The results for $\sigma_{zz\,\text{impurity}}$ and g fitting parameters are presented in Table S1. Compared to pure twisted graphene bilayers[6] and graphite [7], where $g = 0.34$ eV $Å^{-1}$ [3], we observe a significantly smaller value of electron-

phonon coupling $g < 0.1$ eV Å^{-1} and the vertical conductivity, Table S1. This may be due to a notably larger distance between the flakes spaced with pyrene molecules.

S5. On the magnitude of point defects:

The carrier mobility of graphene nanosheets reaches more than 20,000 cm^2 V^{-1} s^{-1} at cryogenic temperatures (Figure 3F). This implies that the number density of point defects in graphene nanosheets should be smaller than $\ell_{\mathrm{mfp}}{}^{-2}$, which is approximately 10^9 cm^{-2}. Raman

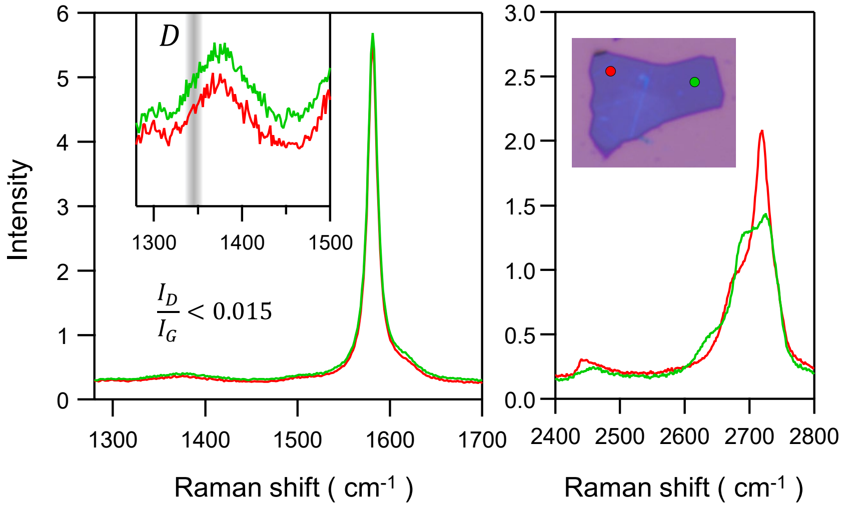

Figure S7. Raman spectra captured from a multi-layered graphene nanosheet (shown in the inset of the right panel). The spots where the spectra are captured are shown in a micrograph. The laser energy was 2.33 eV focused on the samples using a ×100 objective. The inset in the left panel shows the spectrum around the D band region. The gray region indicates the Raman shift corresponding to the D band.

spectroscopy has been widely used to quantify the amount of such defects in graphene and graphite, often by studying the ratio of the D to G bands.[8]

Figure S7 shows the Raman spectrum captured from nanosheets made from high-quality graphite. As discussed in our previous work,[2] the presence of sulfonated pyrene (s-Py) in the nanosheets interferes with the D band signal from possible point defects in the sample. A

weak, broad peak is observed at around 1375 cm^{-1}, originating from s-Py molecules.[2] The absence of any pronounced band at 1340 cm^{-1}, where the D peak originated from point-like defects, is expected, indicating a small number density of point-like defects. Based on the intensity of the spectrum around this energy, we expect $\frac{I_D}{I_G}$ to be smaller than 0.015, which is equivalent to defect density smaller than $3.4 \pm 0.9 \times 10^9$ cm^{-2}.[8]

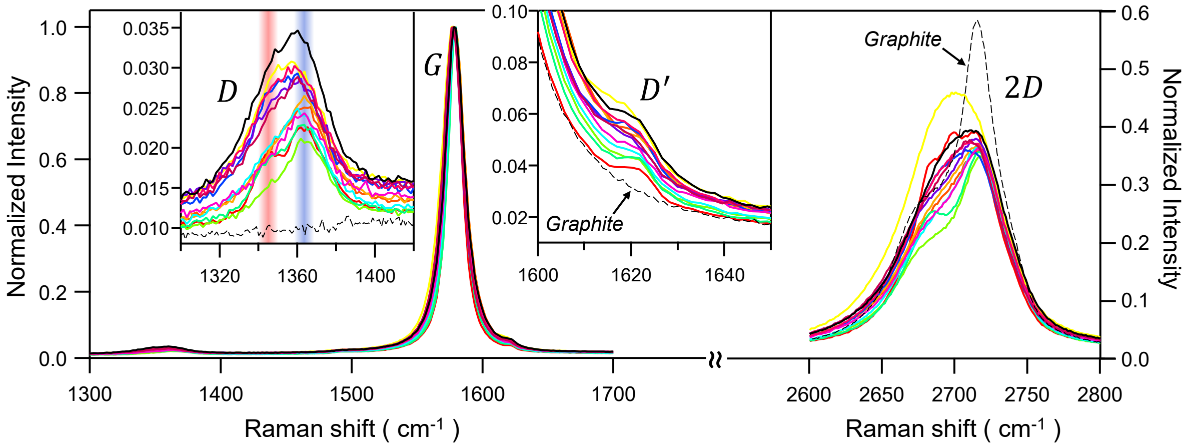

Figure S8. Raman spectra captured from 13 different spots of a graphene laminate annealed at 1500 °C made from NGS graphite crystals. The dashed line shows the graphite spectrum. The D band often showed an asymmetric feature, a characteristic feature of intact graphite and graphene edges.[9]

The Raman peaks due to s-Py disappeared after annealing the laminates above nearly 1000 °C. We often detected weak D bands in the Raman spectrum captured from annealed laminates. However, it originates from edge defects rather than point-like defects. Given that the optical penetration depth for graphite (~60 nm at 532 nm) is much larger than the nanosheet thickness (~4 nm), it is likely that the spectrum is not from the top layer but a few nanosheets combined. In the case of laminates made from NGS graphite (Figure S8), we often observed an asymmetric D band ascribed to the edge defects of graphene or graphite.[9]

In addition, D` band around 1620 $cm^{-1}$ was always observed (Figure S8), and the intensity ratio of D to D` band was also consistent with the edge defect type.[10] We, therefore, conclude that the point-like defect is negligible in our samples, which is consistent with the high mobility of nanosheets at cryogenic temperatures.

S8. Elemental composition of the laminates:

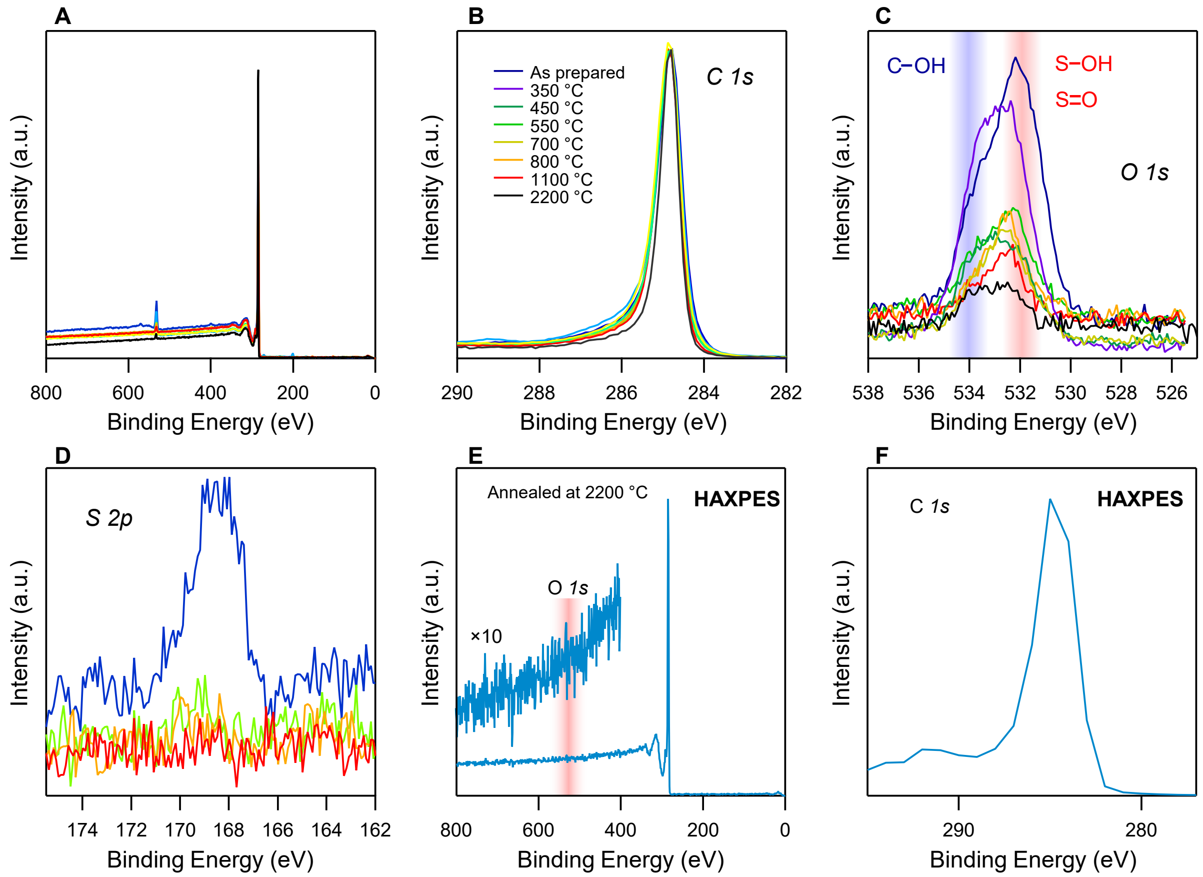

Figure S9. Elemental analysis of graphene laminates. Panels (**A-D**) show the XPS spectra of laminates annealed at different temperatures. Panels (**E**) and (**F**) show the HAXPES spectrum of the laminate annealed at 2200 °C. The oxygen content was below the detection limit. The C 1s peak shows a distinct graphitic structure.

XPS analysis showed that the as-prepared sample contains 0.9, 6.3 and 92.8 atomic percent sulfur, oxygen and carbon, respectively (Figure S9). The sulfur signal is most likely from

tetra-sulfonated pyrene ($C_{16}H_{10}O_{12}S_4$). Upon annealing, most of the sulfur evaporated through de-sulfonating of s-Py. Indeed, this is consistent with the disappearance of the sulfur peak in the XPS spectra of annealed samples (Figure S9D).

As every sulfonated group contains one sulfur and three oxygen atoms, reduction in oxygen content is also consistent with de-sulfonating s-Py upon annealing (Figure S9C). XPS analysis of laminates annealed at 2200 °C showed 0.92 atomic percent oxygen. We ran hard X-ray photoelectron spectroscopy (HAXPES) on the same sample to rule out the oxygen associated with the surface. The oxygen content was below the detection limit (Figures S9E and S9F). This corresponds to a carbon content of 100.00%. This is consistent with the high carrier mobility in our samples.

S9. Observation of weak localization in graphene laminates.

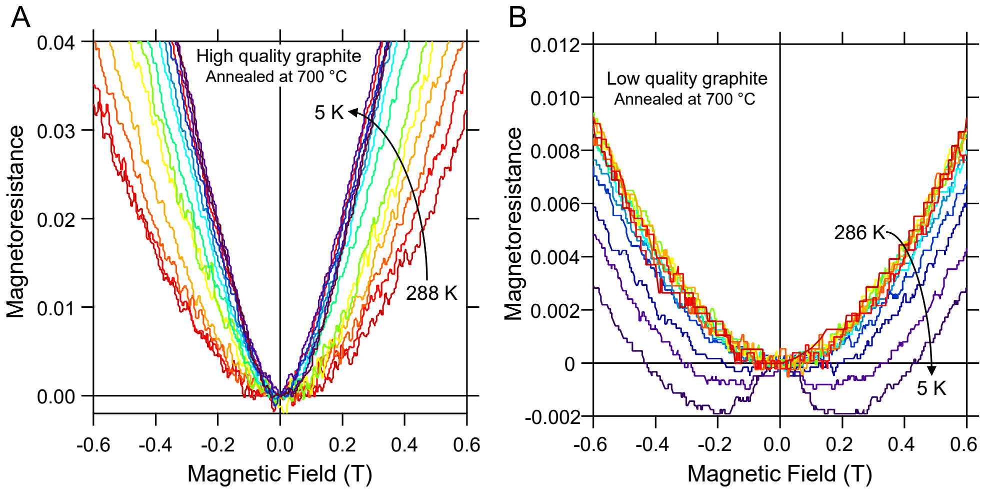

Figure S10. Low-field magnetoresistance for laminates of different quality. (**A**) The magnetoresistance of the laminates produced using high-quality graphite (Graphenium flakes, NGS) was measured at different temperatures. The magnetoresistance remains positive at all temperatures. (**B**) The magnetoresistance of the laminates fabricated using Ashbury graphite. At temperatures lower than 15 K, the magnetoresistance exhibits the negative magnetoresistance ia range around zero magnetic fields (± 0.2 T) typical of weak localization.

The laminates produced using NGS graphite, Figure S10A, have consistently demonstrated temperature-dependent carrier mobility typical for conductors with a low disorder where the electron-phonon scattering dictates the transport properties at higher temperatures. The sign of the magnetoresistance for these samples was always positive regardless of the ambient temperature and postprocessing annealing. Relatively lower transport quality was obtained from the laminates produced using Ashbury graphite, Figure S10B. In this case, the carrier mobility was found to be less sensitive to the ambient temperature as expected for more disordered materials. Also, the transport contained signatures of weak localization seen as negative magnetoresistance at low temperatures previously observed in printed graphene networks.[11]

S10. A summary of literature data on thermoelectric properties of printed graphene films and related materials.

Table S2. Summary of the Seebeck coefficient values obtained from various printed graphene films or other related materials available from the literature. All data points are taken at approximately 300 K and compared against the result shown in Figure 4E.

Material	Seebeck coefficient at ~ 300 K (μV K^{-1})
Partially reduced graphene oxide using hydroiodic[12]	12.5
Graphite traces from commercial pencils[13]	7.4
Pristine CVD graphene and graphene doped with 2-(9-Oxoxanthen-2-yl)propionic acid[14]	80
Reduced graphene oxide and platinum[15]	29.9
CVD graphene[16]	39
$Ti_3C_2T_x$ MXene and liquid phase exfoliated graphene[17]	53.6
titanium carbide (MXene)-silver nanowire-PEDOT:PSS-tellurium nanowire[18]	25.3
Silver-liquid phase exfoliated graphene[19]	55.4
Gold-indium tin oxide[20]	55.64

PEDOT:PSS- carbon nanotubes composites[21]	31
metal–organic framework-micro structured mixed cellulose[22]	57.1
Graphene-PDMS[23]	35.2
MXene decorated chitosan activation of polydimethylsiloxane foam[24]	5
Acid-assisted exfoliated graphene (this work)	49.9

References

(1) Zou, K.; Hong, X.; Zhu, J. Effective Mass of Electrons and Holes in Bilayer Graphene: Electron-Hole Asymmetry and Electron-Electron Interaction. *Physical Review B* **2011**, *84* (8), 085408.

(2) Moazzami Gudarzi, M.; Asaad, M.; Mao, B.; Pinter, G.; Guo, J.; Smith, M.; Zhong, X.; Georgiou, T.; Gorbachev, R.; Haigh, S. J.; et al. Chlorosulfuric Acid-Assisted Production of Functional 2d Materials. *npj 2D Materials and Applications* **2021**, *5* (1), 35.

(3) Perebeinos, V.; Tersoff, J.; Avouris, P. Phonon-Mediated Interlayer Conductance in Twisted Graphene Bilayers. *Physical Review Letters* **2012**, *109* (23), 236604.

(4) Nicklow, R.; Wakabayashi, N.; Smith, H. G. Lattice Dynamics of Pyrolytic Graphite. *Physical Review B* **1972**, *5* (12), 4951-4962.

(5) Ochoa, H.; Castro, E. V.; Katsnelson, M.; Guinea, F. Temperature-Dependent Resistivity in Bilayer Graphene Due to Flexural Phonons. *Physical Review B* **2011**, *83* (23), 235416.

(6) Kim, Y.; Yun, H.; Nam, S.-G.; Son, M.; Lee, D. S.; Kim, D. C.; Seo, S.; Choi, H. C.; Lee, H.-J.; Lee, S. W.; et al. Breakdown of the Interlayer Coherence in Twisted Bilayer Graphene. *Physical Review Letters* **2013**, *110* (9), 096602.

(7) Koren, E.; Leven, I.; Lörtscher, E.; Knoll, A.; Hod, O.; Duerig, U. Coherent Commensurate Electronic States at the Interface between Misoriented Graphene Layers. *Nature Nanotechnology* **2016**, *11* (9), 752-757.

(8) Cançado, L. G.; Jorio, A.; Ferreira, E. H. M.; Stavale, F.; Achete, C. A.; Capaz, R. B.; Moutinho, M. V. O.; Lombardo, A.; Kulmala, T. S.; Ferrari, A. C. Quantifying Defects in Graphene Via Raman Spectroscopy at Different Excitation Energies. *Nano Letters* **2011**, *11* (8), 3190-3196.
(9) Cançado, L. G.; Pimenta, M. A.; Neves, B. R. A.; Dantas, M. S. S.; Jorio, A. Influence of the Atomic Structure on the Raman Spectra of Graphite Edges. *Physical Review Letters* **2004**, *93* (24), 247401. Li, Q.-Q.; Zhang, X.; Han, W.-P.; Lu, Y.; Shi, W.; Wu, J.-B.; Tan, P.-H. Raman Spectroscopy at the Edges of Multilayer Graphene. *Carbon* **2015**, *85*, 221-224.
(10) Eckmann, A.; Felten, A.; Mishchenko, A.; Britnell, L.; Krupke, R.; Novoselov, K. S.; Casiraghi, C. Probing the Nature of Defects in Graphene by Raman Spectroscopy. *Nano Letters* **2012**, *12* (8), 3925-3930.
(11) Cottam, N. D.; Wang, F.; Austin, J. S.; Tuck, C. J.; Hague, R.; Fromhold, M.; Escoffier, W.; Goiran, M.; Pierre, M.; Makarovsky, O.; et al. Quantum Nature of Charge Transport in Inkjet-Printed Graphene Revealed in High Magnetic Fields up to 60t. *Small* **2024**, 2311416. Calabrese, G.; Pimpolari, L.; Conti, S.; Mavier, F.; Majee, S.; Worsley, R.; Wang, Z.; Pieri, F.; Basso, G.; Pennelli, G.; et al. Inkjet-Printed Graphene Hall Mobility Measurements and Low-Frequency Noise Characterization. *Nanoscale* **2020**, *12* (12), 6708-6716.
(12) Barwich, S.; Medeiros de Araújo, J.; Rafferty, A.; Gomes da Rocha, C.; Ferreira, M. S.; Coleman, J. N. On the Relationship between Morphology and Conductivity in Nanosheet Networks. *Carbon* **2021**, *171*, 306-319.
(13) Mulla, R.; Dunnill, C. W. Single Material Thermocouples from Graphite Traces: Fabricating Extremely Simple and Low Cost Thermal Sensors. *Carbon Trends* **2021**, *4*, 100077.

(14) Kirihara, K.; Okigawa, Y.; Ishihara, M.; Hasegawa, M.; Mukaida, M.; Horike, S.; Wang, Y.; Wei, Q. Transparent Patternable Large-Area Graphene P–N Junctions by Photoinduced Electron Doping. *ACS Applied Materials & Interfaces* **2024**, *16* (1), 1198-1205.

(15) Wang, C.; Zhang, Y.; Han, F.; Jiang, Z. Flexible Thermoelectric Type Temperature Sensors Based on Graphene Fibers. In *Micromachines*, 2023; Vol. 14.

(16) Harzheim, A.; Könemann, F.; Gotsmann, B.; van der Zant, H.; Gehring, P. Single-Material Graphene Thermocouples. *Advanced Functional Materials* **2020**, *30* (22), 2000574.

(17) Saeidi-Javash, M.; Du, Y.; Zeng, M.; Wyatt, B. C.; Zhang, B.; Kempf, N.; Anasori, B.; Zhang, Y. All-Printed MXene–Graphene Nanosheet-Based Bimodal Sensors for Simultaneous Strain and Temperature Sensing. *ACS Applied Electronic Materials* **2021**, *3* (5), 2341-2348.

(18) Li, F.; Liu, Y.; Shi, X.; Li, H.; Wang, C.; Zhang, Q.; Ma, R.; Liang, J. Printable and Stretchable Temperature-Strain Dual-Sensing Nanocomposite with High Sensitivity and Perfect Stimulus Discriminability. *Nano Letters* **2020**, *20* (8), 6176-6184.

(19) Juntunen, T.; Jussila, H.; Ruoho, M.; Liu, S.; Hu, G.; Albrow-Owen, T.; Ng, L. W. T.; Howe, R. C. T.; Hasan, T.; Sun, Z.; et al. Inkjet-Printed Large-Area Flexible Few-Layer Graphene Thermoelectrics. *Advanced Functional Materials* **2018**, *28* (22), 1800480.

(20) Bappy, M. O.; Jiang, Q.; Atampugre, S.; Zhang, Y. Aerosol Jet Printing of High-Temperature Bimodal Sensors for Simultaneous Strain and Temperature Sensing Using Gold and Indium Tin Oxide Nanoparticle Inks. *ACS Applied Nano Materials* **2024**, *7* (8), 9453-9459.

(21) He, X.; Hao, Y.; He, M.; Qin, X.; Wang, L.; Yu, J. Stretchable Thermoelectric-Based Self-Powered Dual-Parameter Sensors with Decoupled Temperature and Strain Sensing. *ACS Applied Materials & Interfaces* **2021**, *13* (50), 60498-60507.

(22) Li, Y.; Wang, R.; Wang, G.-E.; Feng, S.; Shi, W.; Cheng, Y.; Shi, L.; Fu, K.; Sun, J. Mutually Noninterfering Flexible Pressure–Temperature Dual-Modal Sensors Based on Conductive Metal–Organic Framework for Electronic Skin. *ACS Nano* **2022**, *16* (1), 473-484.

(23) Wang, Y.; Wu, H.; Xu, L.; Zhang, H.; Yang, Y.; Wang, Z. L. Hierarchically Patterned Self-Powered Sensors for Multifunctional Tactile Sensing. *Science Advances 6* (34), eabb9083.

(24) Gao, F.-L.; Liu, J.; Li, X.-P.; Ma, Q.; Zhang, T.; Yu, Z.-Z.; Shang, J.; Li, R.-W.; Li, X. $Ti_3C_2T_x$ Mxene-Based Multifunctional Tactile Sensors for Precisely Detecting and Distinguishing Temperature and Pressure Stimuli. *ACS Nano* **2023**, *17* (16), 16036-16047.
